# Distalization of perianal fistulas after loose silicone seton drainage is a myth

**DOI:** 10.1007/s10151-023-02882-3

**Published:** 2023-12-14

**Authors:** Carolien Verkade, G. Fiek A. J. B. van Tilborg, Jasper Stijns, Daria K. Wasowicz, David D. E. Zimmerman

**Affiliations:** 1 Colorectal Research Group, Department of Surgery, Elisabeth-TweeSteden Hospital, Tilburg, The Netherlands; 2 Department of Radiology, Elisabeth-TweeSteden Hospital, Tilburg, The Netherlands; 3 Department of Surgery, University Hospital Brussels, Brussels, Belgium

**Keywords:** Rectal fistula, Fistula-in-ano, Complex perianal fistula, Seton, Magnetic resonance imaging, Distalization, Distalisation, Outward migration

## Abstract

**Background:**

It is often stated that loose seton drainage results in distal migration of a fistula tract in perianal fistula. The aim of the present study was to assess this distalization of trans- and suprasphincteric perianal fistulas after a silicone seton has been inserted.

**Methods:**

Consecutive patients who underwent loose seton placement for the management of a transsphincteric or suprasphincteric fistula between January 2016 and December 2021 with a pre- and postoperative magnetic resonance imaging (MRI) were included in the present retrospective study. The height of the external anal sphincter (EAS) and the level of penetration of perianal fistula through the EAS or puborectal muscle (PRM) were determined on MRI. Primary outcome was migration of the fistula tract through the EAS and PRM.

**Results:**

Thirty-eight patients with perianal fistulas were included. Median height of the EAS was 28 (IQR 25–34) mm before seton placement and 27 (IQR 24–33) mm afterward. Median level of perforation was 32 (IQR 17–40) mm before seton placement and 28 (IQR 17–40) mm afterward (*p* = 0.37). One fistula (3%) was downgraded from mid to low transsphincteric and was laid open after 14.9 months of loose seton drainage.

**Conclusions:**

No statistically significant distalization of complex fistula tracts after loose silicone seton drainage was found. Some complex fistulas may downgrade to a less complex fistula after long-term seton drainage. However, loose silicone seton drainage should not be offered to patients as a treatment option to downgrade a complex fistula to a simple one or even have the hope to heal it.

## Introduction

Cutting setons have been used since Hippocrates. The cutting seton technique is based on the assumption that it causes an inflammatory reaction which stimulates fibrosis around the seton, preventing the sphincter from retraction when slowly divided. Unlike ‘laying open’ of the fistula tract, this technique should preserve optimal continence. Today, the use of cutting setons is controversial because of the significant patient discomfort after tightening and the wide range of reported incontinence rates [1].

Alternatively, a seton can be placed through the fistula tract and left loose without tightening, facilitating drainage. This loose seton technique is often applied prior to definitive repair and is also used as a palliative measure aimed to control symptoms and avoid formation of new abscesses. It is believed that long-term loose seton drainage causes distal migration of the fistula tract [2]. Studies that report on this supposed phenomenon are scarce.

The aim of the present study is to assess the occurrence of distalization of trans- and suprasphincteric perianal fistulas after loose silicone seton drainage.

## Methods

Consecutive patients undergoing loose silicone seton placement for the management of a trans- or suprasphincteric fistula between January 2016 and December 2021 who met the inclusion criteria were studied retrospectively.

Inclusion criteria were magnetic resonance imaging (MRI) within 3 months before loose seton placement and MRI after loose seton placement. In our centre MRI is routinely performed for assessment of perianal fistulas. Exclusion criteria were seton replacement after seton loss instead of primary seton placement and surgery between seton placement and MRI.

**Fig. 1:**
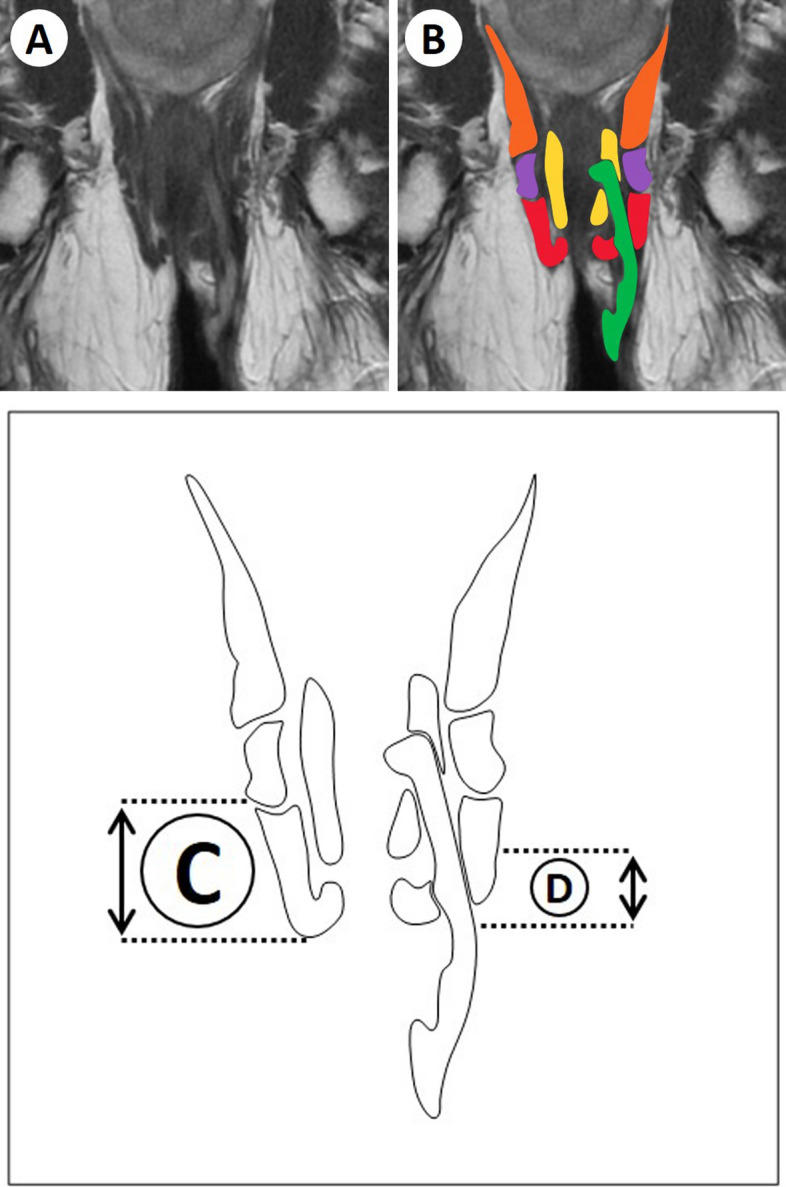
Measuring external anal sphincter  length and level of penetration. **A** Coronal sequence fistula MRI. **B** Perianal anatomy on MRI: green = fistula, yellow = internal anal sphincter, red = external anal sphincter, purple = puborectal muscle, orange = levator ani muscles. **C** Length of the external anal sphincter. **D** Level of penetration

The height of the external anal sphincter (EAS) and the level of penetration of perianal fistula through the EAS or puborectal muscle (PRM) were determined on pre- and postoperative MRI (Fig. 1). The level of penetration was defined as the most cranial part of penetration through the EAS or PRM. All measurements were prospectively performed by a radiologist with special interest in perianal fistulas. The radiologist was blinded to the MRI sequence.

Primary outcome was migration of the fistula tract through the EAS and PRM. The level of penetration of the fistula was divided by the EAS height to determine the proportion of muscle involved. Migration was determined by comparing the proportion of involved muscle before and after seton placement.

Data collected included demographics, previous fistula surgery, fistula characteristics and time to MRI.

### Statistical analysis

Data were analysed using the Statistical Package for Social Sciences (SPSS), version 25.0 (IBM Corp., Armonk, NY, USA). Non-parametric data are given as median with interquartile range (IQR). The Wilcoxon signed rank test was used to compare non-parametric data. Categorical data are given in absolute numbers with percentages. A *p*-value < 0.05 was considered statistically significant.

## Results

Thirty-eight patients [male:female = 29:9, median age 40.1 (IQR 29.6–53.7) years] were included. Ten patients (26%) had Crohn’s disease. Twelve patients (32%) had previously undergone loose seton drainage. Two patients (5%) presented with multiple complex fistula tracts (respectively 2 transsphincteric tracts and 2 suprasphincteric tracts). Loose silicone setons were placed in all tracts. All 40 setons were knot-free Comfort Drains (AMI, Feldkirch, Austria).

Median height of the EAS was 28 (IQR 25–34) mm before seton placement and 27 (IQR 24–33) mm after seton placement. Most fistulas (*n* = 24, 60%) penetrated the PRM. Six fistulas (15%) penetrated the EAS and the PRM. Median level of perforation was 32 (IQR 17–40) mm before seton placement and 28 (IQR 17–40) mm after seton placement. The median proportion of involved EAS was 1.1 before and after seton placement. The ratio exceeded one since most fistulas were suprasphincteric. No significant migration of the fistula tract through the EAS and PRM was detected (*p* = 0.37). When analysed separately, no statistically significant migration was found for either fistulas of cryptoglandular origin or fistulas related to Crohn’s disease (respectively *p* = 0.90 and *p* = 0.15).

Median duration of seton drainage to MRI was 5.7 (IQR 3.0–9.7) months. One fistula (3%) was downgraded from mid to low transsphincteric and was laid open after 14.9 months of loose seton drainage.

## Discussion

In the present study, we assessed distal migration of complex fistulas through the external anal sphincter (EAS) and puborectal muscle (PRM) after loose silicone seton drainage. Distalization of the fistula tract was determined by calculating the proportion of muscle involved on magnetic resonance imaging (MRI). To the best of our knowledge, this study is the first to assess distalization in an MRI-controlled manner.

Fistulas of cryptoglandular origin and those associated with Crohn’s disease were assessed in the present study. Both groups received identical setons and were assessed in the same manner. While differentiating between aetiologies is essential for treatment, we postulated that it was not of interest for determining distalization.

Seton techniques are divided into two main categories: the cutting seton and the loose seton. Cutting setons are intended to gradually cut through the enclosed sphincter muscle to heal the fistula with preservation of faecal continence. Reported faecal incontinence rates after cutting seton treatment range widely with an average rate of 12% [1]. Patients with complex perianal fistulas have a higher risk of faecal incontinence after cutting seton treatment [1]. Continuous sphincter division can cause severe pain, especially after tightening. Many European surgeons have abandoned the cutting seton technique [3]. The American Society of Colon and Rectal Surgeons (ASCRS) Clinical Practice Guidelines still state that cutting setons may be used selectively in the management of complex anal fistulas [4]. However, the recommendation was recently changed from grade 2B in 2016 to grade 2C [4]. The use of cutting setons is not recommended by the European Society of Coloproctology, the German S3 guideline and the Dutch national guideline for perianal fistulas [5–7].

The loose seton technique facilitates draining by keeping the fistula tract open, which should prevent abscess formation. Loose setons are also referred to as non-cutting setons since they are not intended to cut through the enclosed tissue. However, it is widely believed that loose setons cause slow distal migration of the fistula tract. It has been postulated that bowel movements cause moderate pull on a loose seton, resulting in the fistula gradually but continually becoming shorter [8, 9]. This suggests long-term seton drainage can downstage a complex perianal fistula, allowing secondary fistulotomy. Some surgeons also tell their patients that a loose seton might distalise over time [10]. The ASCRS manual even mentions this option [2]. In our opinion, it is impossible to estimate the exact percentage of involved EAS without additional imaging by either MRI or endoanal ultrasound. These measurements are seldom performed and have never been reported. Therefore, it is likely that some patients undergo fistulotomy after seton drainage who may have been amenable to safe fistulotomy in the first place.

In the present study, only 1 of the 40 fistulas treated with a loose silicone seton was downgraded from a mid-transsphincteric to a low-transsphincteric fistula after > 1 year of seton drainage. A relatively small amount of EAS was involved in this transsphincteric fistula and the tract migrated only a few millimetres after a year of seton drainage to become low transsphincteric. It could be argued that this particular fistula might have been treated by simple fistulotomy in the first place. Generally, surgeons advocate that fistulas traversing the lower third of the external anal sphincter can be safely laid open. This theorem has rarely been assessed in a controlled setting. The only exception is the study by Garces-Albir et al. who performed pre- and postoperative endoanal sonography to assess the amount of sphincter cut during fistulotomy [11]. Interestingly, they conclude that possibly much more than one-third may be safely cut. Even though most surgeons will be justifiably hesitant about cutting more than one-third, good functional outcome has been described by other authors as well in less well-controlled settings [12]. In our opinion, it is likely that some surgeons and patients change their perspective after long-term seton drainage and are willing to accept cutting more sphincter than they initially were, yielding acceptable outcomes even if substantial parts of the external anal sphincter are cut.

Seventy-seven per cent of surgeons will use a draining seton as part of a staged surgical approach [3]. Limited data are currently available to assess the value of this strategy in different applications. Some data support this use prior to flap repair; application of draining seton prior to LIFT is controversial [3]. Most experts advocate the use of a draining seton to improve quality of life and even as a “palliative measure” for patients who are unwilling or unable to undergo sphincter cutting procedures. Current data suggest that inserting a silicone draining seton to downgrade the fistula is not a valid strategy and should not be offered to the patient as such. Moreover, restraint should be exercised in counselling patients that insertion of loose draining setons may lead to simplifying the fistula and in this way creating additional treatment options.

A variety of materials is used for loose seton drainage. As stated earlier, the type of material might affect the migration distance of a loose seton [13, 14]. Two studies reported extensive outward migration of loose silk setons [9, 15]. In one study patients were instructed to rotate their loose silk seton daily to promote distalization of the fistula tract [15]. Another study used a nonabsorbable braided suture of medium thickness with the ends tied loosely together to treat intersphincteric and low transsphincteric fistulas [8]. Like the loose silk setons, these setons migrated outward after prolonged seton drainage. A recent international survey on seton material showed that silicone is the most utilized material [3]. Bellini et al. reported on the outward migration of the internal orifice after long-term loose, mainly vessel-loop seton drainage [14]. Migration of the internal orifice was measured using an anoscope and flexible ruler. A limited but statistically significant extent of outward migration of the internal orifice was found after a mean seton duration of 18.8 months. The authors mentioned an epithelial covering of the migration path with a distinct step off. In our tertiary referral hospital we come across a variety of materials and techniques used for seton drainage in referred patients, but we have never noticed such a migration path for any of these setons.

In the present study we used MRI scans to assess distalization. In our clinic we do not have any experience with endoanal ultrasound. However, it is likely that similar measurements can be obtained in 3D reconstructions of the fistula and sphincter complex. We considered repetitive measurements to assess interobserver variability but did not do so because of the extremely time-consuming nature of this additional task.

No data are available comparing the comfort of different seton materials. If silk setons are as comfortable as silicone setons, selecting silk setons may be preferable as limited data suggest that this material may promote distalization [9, 15]. Investigating and comparing different materials used for seton drainage would be an interesting avenue of research. Moreover, whether or not fistulas can be treated by simple fistulotomy initially should be considered, avoiding the indication of a seton.

## Conclusion

We did not find statistically significant distalization of complex fistula tracts after loose silicone seton drainage. Some complex fistulas might be downgraded to a less complex fistula after long-term seton drainage. However, loose silicone seton drainage should not be offered to patients as a treatment option to downgrade or heal a complex perianal fistula.

## Data Availability

The datasets analysed during the current study are available from the corresponding author on reasonable request.
